# Supplementary figures and images for: Changes in Plasma Itaconate Elevation in Early Rheumatoid Arthritis Patients Elucidates Disease Activity Associated Macrophage Activation
Source: Metabolites. 2020 Jun 10;10(6):241. doi: 10.3390/metabo10060241 (PMC7344783; doi:10.3390/metabo10060241)

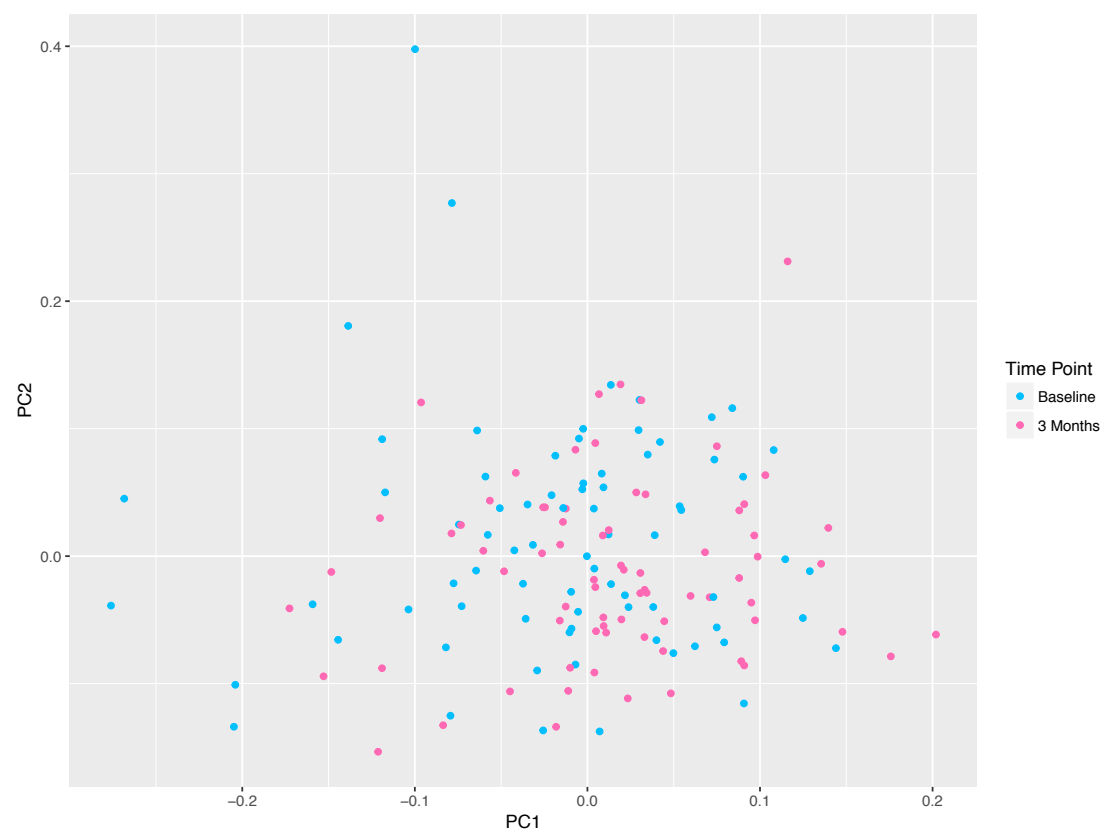

*Figure 1 Principal Component Analysis plot of the peak data at baseline and 3 months*

Supplement: Supplementary file 1 [file metabolites-10-00241-s001.zip › Supp 1 - TaSER_AandR_Metabolomics_Supplementary_Figures.pdf]
